# Susceptibility of Diabetic Mice to Noise Trauma

**DOI:** 10.1155/2018/7601232

**Published:** 2018-02-12

**Authors:** Wook Kyoung Han, Eung Hyub Kim, Sun-Ae Shin, Dong-Sik Shin, Bong Jik Kim, Ah-Ra Lyu, Yong-Ho Park

**Affiliations:** ^1^Department of Otolaryngology-Head and Neck Surgery, College of Medicine, Chungnam National University, Daejeon, Republic of Korea; ^2^Department of Medical Science, College of Medicine, Chungnam National University, Daejeon, Republic of Korea; ^3^Biomedical Convergence Research Center, Chungnam National University Hospital, Daejeon, Republic of Korea; ^4^Brain Research Institute, College of Medicine, Chungnam National University, Daejeon, Republic of Korea

## Abstract

Diabetes can lead to many end-organ complications. However, the association between diabetes and hearing loss is not well understood. Here, we investigated the effect of noise exposure on diabetic mice compared with wild-type mice. Hearing threshold shifts, histopathologic changes in the cochlea, and inflammatory responses were evaluated over time. After noise exposure, more severe hearing threshold shifts, auditory hair cell loss, and synaptopathies were notable in diabetic mice compared with wild-type mice. Moreover, increased inflammatory responses and reactive oxygen species production were observed in the ears of diabetic mice. The results demonstrated that diabetic mice are more susceptible to noise trauma.

## 1. Introduction

Diabetes is a representative metabolic disease that results in many complications [1–3]. In general, patients with diabetes are prone to inflammatory diseases and increased inflammatory responses in many organs [4–11] and are more vulnerable to trauma and tissue injuries in end organs [1, 3, 12–14]. Diabetes per se is regarded as a risk factor of many inflammatory diseases and trauma due to disrupted homeostasis and immune responses [4–6, 9, 11].

Hearing loss is a highly heterogeneous disorder, with multifactorial causes including infections, genetic etiologies, and physical or noise traumas to the inner ear. Hearing loss could occur congenitally or gradually reaching about a half in prevalence for those older than 75 (https://www.nidcd.nih.gov/health/statistics/quick-statistics-hearing). Hearing loss can be divided into sensorineural hearing loss, conductive hearing loss, mixed hearing loss, or neural hearing loss according to the mechanism of hearing loss. Specifically, noise-induced hearing loss is the second most frequent form of sensorineural hearing impairment and noise trauma is a well-studied universal trigger for hearing loss throughout the life with its main pathophysiologic mechanism based on mechanical destruction and metabolic decompensation, resulting in pathologies in ribbon synapses and organ of Corti [15–18]. Given that diabetes is a systemic metabolic disease affecting almost all parts of human body, it would be worth investigating the hearing loss in relation to diabetes.

Although there have been many reports on optic and peripheral neuropathies in diabetes, the association between diabetes and hearing loss is not well understood. Recently, meta-analysis and cohort studies showed that hearing impairment is associated with diabetes and insulin resistance [19–22], putting diabetic patients on an increased risk of future hearing loss.

Thus, we speculated that the cochlea of diabetic mice also responds differently to injuries and trauma such as noise compared with that of wild-type mice, and sensorineural hearing loss may be a delayed complication of diabetes. In this study, we aimed to evaluate the effect of noise trauma as well as the inflammatory responses in diabetic mice compared with wild-type mice via functional and morphologic studies.

## 2. Materials and Methods

### 2.1. Animals and Noise Exposure

All animal experiments were approved by the Chungnam National University Animal Experiment Committee (CNU00859). For this study, 30 C57BL/6J db/db (^++^) mice and 30 age-matched wild-type mice at 7 weeks of age were used. In each group, 25 mice were exposed to noise, and the remaining five were not exposed to noise, serving as controls. The mice were exposed to broadband noise (250 to 8 kHz) at 116 dB SPL for 1 h in an acoustically insulated reverberation chamber. The noise signals were routed through a computer and amplifier (INTER-M R300 Plus power amplifier, Canford Audio PLC, Washington, UK) to a loudspeaker (ElectroVoice DH1A-WP, Sonic Electronix Inc., Sylmar, CA, USA). The noise level was measured using a sound level meter (B&K type 2250, Brüel & Kjaer, Naerum, Denmark), sound calibrator (B&K type 4231, Brüel & Kjaer), and a condenser microphone (B&K type 4189, Brüel & Kjaer).

### 2.2. Auditory Brainstem Response (ABR)

Auditory brainstem response (ABR) was measured as previously reported [23]. The ABRs were recorded prior to, just after, and at 1 day, 1 week, and 2 weeks after noise exposure. Threshold shift was defined as the difference between the before- and after-noise exposure values. A positive threshold shift indicated an elevation of the auditory threshold.

### 2.3. Tissue Preparation and Immunohistochemistry

Animals were sacrificed, and cochlear tissues were obtained to assess survival of hair cells, nerve fibers, and the synaptic ribbon. Tissue preparations were performed as previously reported [23]. Auditory hair cells, nerve fibers, and the ribbon synapse were evaluated by incubating the tissues with rabbit anti-myosin VIIA (Proteus BioSciences, Inc., Ramona, CA, USA), mouse anti-NF200 (Novus Biologicals, Littleton, CO, USA), and mouse anti-C-terminal binding protein 2 primary antibodies (BD Biosciences, San Jose, CA, USA), respectively, diluted 1 : 200 in blocking solution overnight at 4°C. After rinsing in PBS for 10 min, the tissues were incubated with an Alexa Fluor 594-conjugated goat anti-rabbit secondary antibody (Molecular Probes, Eugene, OR, USA) or Alexa Fluor 488-conjugated goat anti-mouse secondary antibody (Molecular Probes) diluted 1 : 200 in PBS for 30 min. After another rinse in PBS for 10 min, the specimens were further dissected to separate individual cochlear turns and mounted on glass slides using Crystal Mount (Biomeda, Foster City, CA, USA). The specimens were observed under an epifluorescence microscope (Zeiss Axio Scope A1; Zeiss, Oberkochen, Germany) with a digital camera, and the number of stained hair cells per 100 *μ*m of tissue was counted.

### 2.4. Quantitative Reverse-Transcription Polymerase Chain Reaction (qRT-PCR)

**Figure 1:**
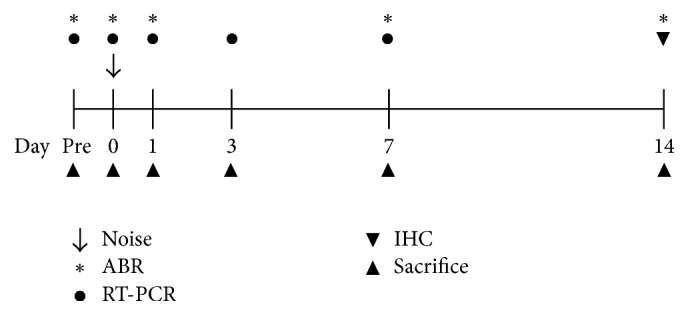
Schematic time course of the experiments. Auditory brainstem response (ABR) thresholds were measured prior to; immediately after; and at 1 day, 1 week, and 2 weeks after noise exposure. Reactive oxygen species and inflammatory responses were evaluated by quantitative reverse-transcription polymerase chain reaction (qRT-PCR) prior to; immediately after; and at 1 day, 3 days, and 1 week after noise exposure. The numbers of hair cells and inner hair cell synapses were assessed at 2 weeks after noise exposure. Five diabetic and 5 wild-type animals were used for each time point.

To compare the inflammatory responses and production of reactive oxygen species (ROS) between groups, 5 diabetic and 5 wild-type animals were sacrificed at each time point after noise exposure. qRT-PCR was conducted to measure the expression of interleukin-1*β* (IL-1*β*), IL-6, nitric oxide synthase 2 (NOS2), tumor necrosis factor-*α* (TNF-*α*), and cyclooxygenase 2 (COX2), as indicators of the inflammatory response. Heme oxygenase-1 (HO-1), superoxide dismutase 1 (SOD1), catalase, and nuclear respiratory factor 1 (NRF1), as oxidative stress and ROS markers, were also measured. qRT-PCR was performed as previously reported [23]. The primers used are presented in Table 1. The time lines for all experiments are shown in Figure 1.

### 2.5. Image Processing and Statistical Analysis

Adjustment of image contrast, image superimposition, and colorization of monochrome fluorescence images were performed using Adobe Photoshop (version 7.0). Statistical analysis was performed using GraphPad Prism (version 3.02, San Diego, CA, USA) and SPSS (version 16.0, SPSS Inc., Chicago, IL, USA). ABR threshold shifts and the levels of ROS and inflammatory cytokines measured in each group were compared before and after noise exposure using one-way repeated measures analysis of variance (ANOVA), and the differences between groups at each time point were compared using one-way ANOVA. The numbers of surviving hair cells and synapses between groups were compared using the Kruskal–Wallis test. *p* values < 0.05 were considered significant.

## 3. Results

### 3.1. ABR Threshold Shifts

**Figure 2:**
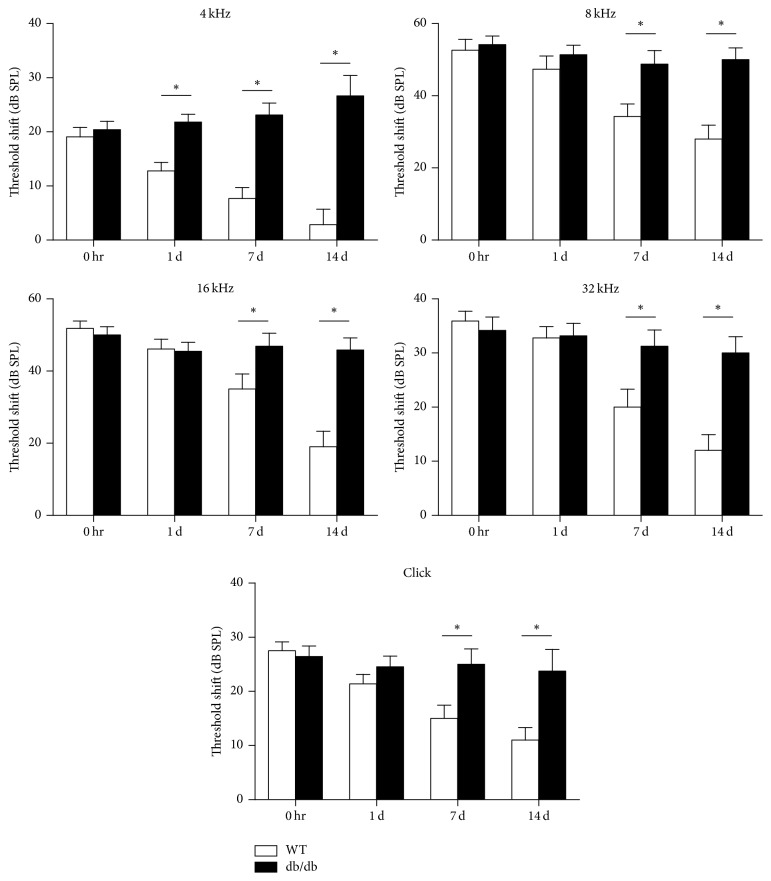
ABR threshold shifts immediately after noise exposure. ABR threshold shifts were greater in db/db mice compared with wild-type mice at 1 and 2 weeks after noise exposure at all frequencies evaluated. ^*∗*^*p* < 0.05.

The ABRs were recorded prior to; immediately after; and at 1 day, 1 week, and 2 weeks after noise exposure. While the ABR threshold shifts recovered partially with time in wild-type mice, they did not recover in db/db mice until 2 weeks after noise exposure. ABR threshold shifts were significantly greater at 1 and 2 weeks after noise exposure in db/db mice compared with wild-type mice (*p* < 0.05) (Figure 2). These results suggested that db/db mice ears were more damaged and susceptible by noise exposure compared to wild-type mice.

### 3.2. Loss of Auditory Hair Cells

**Figure 3:**
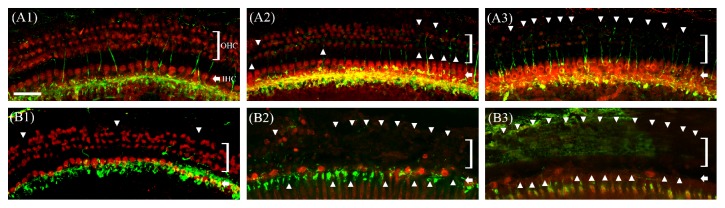
Whole mounts of the auditory epithelium from wild-type (A1, A2, and A3) and db/db mice (B1, B2, and B3) at 2 weeks after noise exposure. Tissues were stained for myosin VIIa (red) to identify the hair cells and for NF200 (green) to identify nerve fibers and then observed by epifluorescence microscopy. Hair cell loss was more severe in the middle (B2) and basal turns (B3) of the db/db compared with wild-type mice (A2 and A3). (A1) and (B1): apical turn; (A2) and (B2): middle turn; A3 and B3: basal turn; OHC: outer hair cell; IHC: inner hair cell; scale bar = 30 *μ*m.

**Figure 4:**
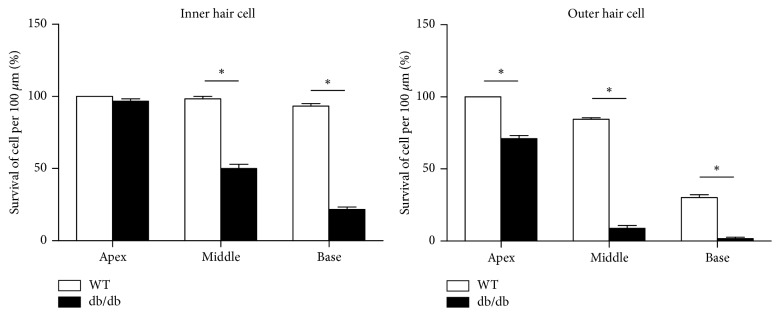
Surviving hair cell counts after surgery. Greater preservation of the outer hair cells was observed in all cochlear turns and of the inner hair cells in the middle and basal turns of wild-type mice compared with db/db mice. ^*∗*^*p* < 0.05.

Two weeks after noise exposure, almost all outer hair cells in the basal turn were destroyed in both db/db (Figure 3(B3)) and wild-type (Figure 3(A3)) mice, but greater preservation of the inner hair cells was evident in wild-type mice (Figure 3(A3)) compared with db/db mice (Figure 3(B3)). In the middle turn, greater preservation of the outer hair cells was observed in wild-type mice (Figure 3(A2)) compared with db/db mice (Figure 3(B2)). The number of surviving hair cells was also significantly higher in wild-type mice (Figure 4), suggesting that the auditory hair cells in db/db mice were more vulnerable to noise trauma.

### 3.3. Loss of Synapses in Inner Hair Cells

**Figure 5:**
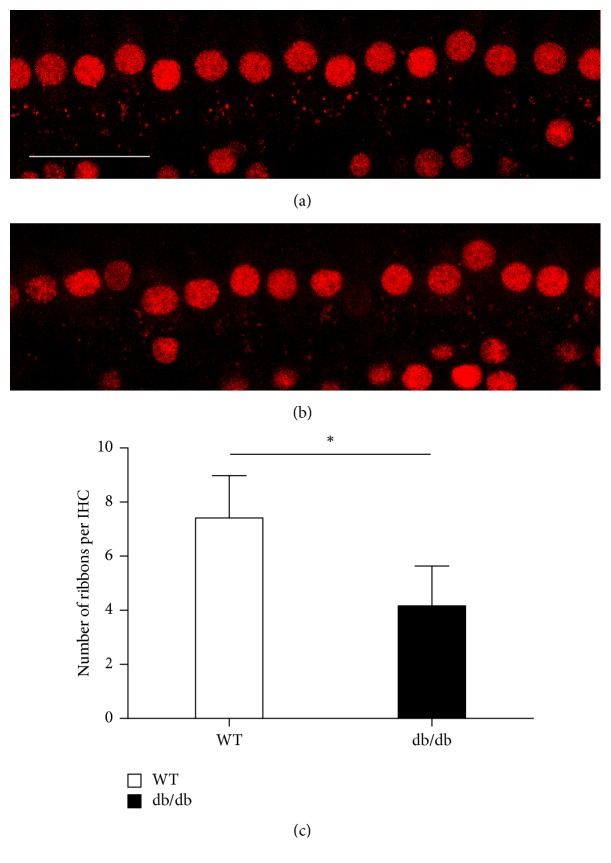
Inner hair cells and their afferent synapses in the middle turn after noise exposure. Tissues were stained for C-terminal binding protein 2 (red) to identify the presynaptic ribbons and then imaged using confocal microscopy. Loss of synapses was more severe in db/db mice (b) compared with wild-type mice (a, c). ^*∗*^*p* < 0.05.

Two weeks after noise exposure, synapse loss in the middle turn of the cochlea was significantly more severe in db/db mice compared with wild-type mice (Figures 5(a), 5(b), and 5(c)). This suggested that, even in surviving inner hair cells, synaptopathies were more severe in db/db mice than in wild-type mice after noise exposure. Synaptopathies in basal turn was not evaluable because almost all hair cells including inner hair cells were lost in the basal turn.

### 3.4. Changes in Markers of Oxidative Stress and ROS

**Figure 6:**
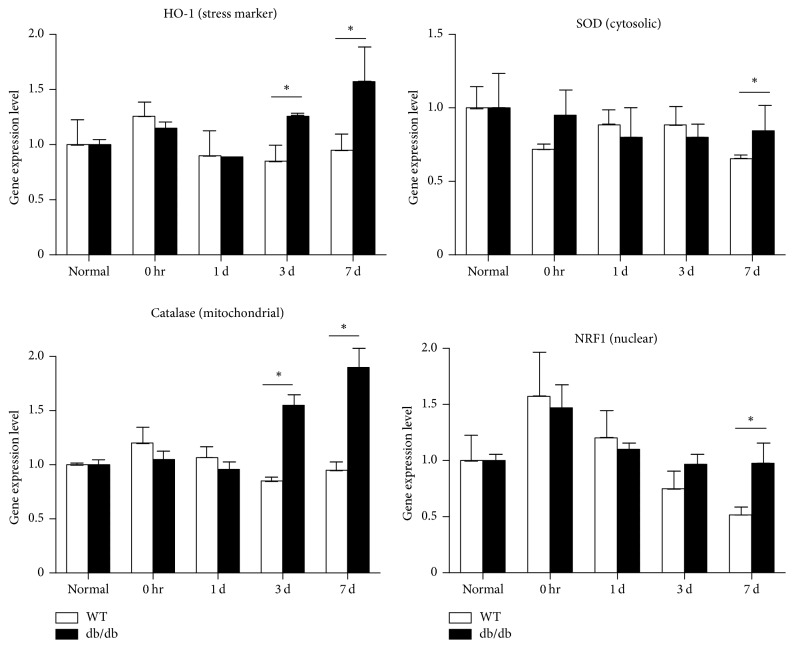
qRT-PCR analysis of reactive oxygen species after noise exposure. HO-1 and catalase levels were significantly increased in db/db compared with wild-type mice at 3 days and sustained until 7 days, after noise exposure. Superoxide dismutase 1 (SOD1) and nuclear respiratory factor 1 (NRF1) levels were significantly increased in db/db mice at 7 days after noise exposure. ^*∗*^*p* < 0.05.

After noise exposure, HO-1 and catalase levels were increased at 3 and 7 days, and SOD1 and NRF1 levels were increased at 7 days in db/db mice compared with wild-type mice. The mild increases in HO-1 and NRF1 levels observed in wild-type mice immediately after noise exposure returned to normal levels with time. These results suggested that ROS production after noise exposure was greater in db/db mice compared with wild-type mice (Figure 6).

### 3.5. Changes in Inflammatory Cytokines

**Figure 7:**
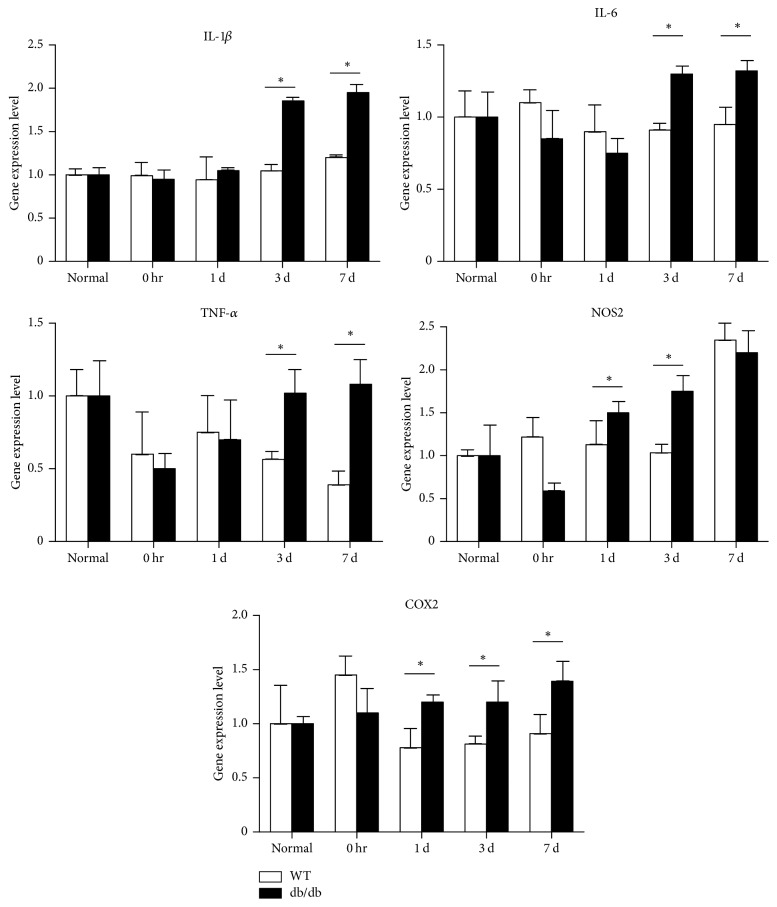
qRT-PCR analysis of inflammatory markers after noise exposure. Significantly increased expression was seen for cyclooxygenase 2 (COX2) at 1, 3, and 7 days, for IL-1*β*, IL-6, and TNF-*α* at 3 days and 1 week, and for nitric oxide synthase 2 (NOS2) at 3 days after noise exposure in db/db compared with wild-type mice. ^*∗*^*p* < 0.05.

IL-1*β*, IL-6, and TNF-*α* levels were significantly increased at 3 and 7 days, NOS2 levels at 1 and 3 days, and COX2 levels at 1, 3, and 7 days after noise exposure in db/db mice compared with wild-type mice. The mild increases in IL-6 and COX2 observed in the wild-type mice immediately after noise exposure returned to normal levels with time (Figure 7). These results suggested that inflammatory responses in the cochlea were more severe in db/db mice than in wild-type mice.

## 4. Discussion

Diabetes can cause many organic complications as a result of neuropathies and angiopathies [1–3, 12]. The association between hearing loss and diabetes is not well known; however, several reports have shown that diabetes is associated with, and a potential risk factor for, hearing loss. Recently, large population studies have revealed that diabetes is an independent risk factor for hearing loss [19, 22, 24]. Kim et al. showed that diabetes was associated with the development of bilateral hearing loss in prospective cohort study [19] and there were reports that showed the association of hearing loss with both type 1 [20] and type 2 diabetes [21]. Furthermore diabetes is also associated with a poor prognosis in terms of recovery of sudden hearing loss [25–30]; Lin et al. revealed that the incidence of sudden hearing loss was 1.54-fold higher in the diabetic group compared with that in the nondiabetic group [27] and sudden hearing loss may be an initial symptom or complication of diabetes [31, 32]. Although the precise etiologies of these associations are not well known, they may involve histopathological changes in the cochlea including hair cells, spiral ganglion neurons (SGNs), and the lateral wall, according to studies in animal models of diabetes [24, 33, 34] and human temporal bone [35–39].

Noise is a common trauma imposed on the ear, and it can induce transient or permanent threshold shifts according to the level and timing of the noise exposure. In this study, we compared the effect of noise on hearing threshold shifts between diabetic and wild-type mice. The results indicated that hearing loss was more severe in diabetic mice compared with wild-type mice. Although we used a transient hearing threshold shift model, the hearing threshold shift did not recover in diabetic mice compared with wild-type mice until 2 weeks after noise exposure and resulted in loss of hair cells and synaptopathies, especially in the middle and basal turns of the cochlea. This coincided well with other reports that streptozocin-induced diabetic mice exhibited greater susceptibility to noise trauma, decreased cochlear blood flow, SGN loss, and failed recovery of ABR threshold shifts and distortion product otoacoustic emissions [13]. In addition, another report showed no recovery of hearing threshold after noise-induced temporary hearing loss in diabetic mice [14].

Noise can induce inflammatory responses in the cochlea, as well as tissue injury and hearing loss [40, 41]. Tan et al. showed that acute and chronic noise exposure could induce the expression of proinflammatory mediators in the cochlea and mediate the recruitment and extravasation of inflammatory cells into the cochlea. As a result, they postulated that cochlear inflammatory response could be induced by noise exposure [40]. Besides, Liu et al. reported the increased level of inducible and endothelial NOS in diabetic rat cochleae, which might be involved in the cochlear functional loss [42]. Thus, in this study, we compared the inflammatory response in the cochlea after noise exposure between diabetic and wild-type mice. As a result, oxidative stress and ROS markers were increased in both diabetic and wild-type mice during the early stage following noise exposure; however, the increased HO-1 level was sustained until 7 days after noise exposure in the diabetic mice, suggesting that ROS production is increased for longer in diabetic compared with wild-type mice.

The inflammatory cytokines IL-1*β*, IL-6, NOS2, TNF-*α*, and COX2 were also significantly increased in diabetic mice compared with wild-type mice. These results indicate an elevated inflammatory response in diabetic mice, which may induce greater oxidative stress and ROS production and, thereby, tissue damage such as hair cell loss and synaptopathies. This result was supported by previous report showing that N-acetylcysteine, a powerful antioxidant, attenuated the degree of noise-induced permanent hearing loss in diabetic rats [43].

In this study, we investigated the effect of noise exposure on diabetic mice compared with wild-type mice. Diabetic mice showed a more severe hearing threshold shift, hair cell loss, and synaptopathies compared with wild-type mice. Increased inflammatory responses and ROS production are possible reasons for these effects in diabetic mice. So we thought that ROS scavengers or anti-inflammatory reagents would be applicable for the prevention of diabetes associated hearing loss.

## 5. Conclusion

Taken together, our study suggests that diabetic mice seem to be more susceptible to noise trauma than wild-type mice. This might lead to more robust hearing loss in diabetic mice, as evidenced by more severe hair cell damage and synaptopathy due to increased inflammatory responses and ROS production in diabetic mice.

## Figures and Tables

**Table 1:** The primer sequences used for quantitative reverse-transcription polymerase chain reaction.

	Forward	Reverse
GAPDH	TGTGTCCGTCGTGGATCTGA	CCTGCTTCACCACCTTCTTGAT
HO-1	CCCACCAAGTTCAAACAGTCT	AGGAAGGGGGTCTTAGCCTC
SOD1	GTATGGGGACAATACACAAGGC	GGCCACCATGTTTCTTAGAGTG
Catalase	TCA GGA TGT GGT TTT CAC TG	GTG TAA AAT TTC ACT GCA AAC
NRF1	GCT GCT GCG TGG CAA CAG	TTG GGT TTG GAG GGT GAG AT
IL-1*β*	TCTTTGAAGTTGACGGACCC	TGAGTGATACTGCCTGCCTG
IL-6	TCGTGGAAATGAGAAAAGAGTTG	AGTGCATCATCGTTGTTCATACA
TNF-*α*	CTGAGGTCAATCTGCCCAAGTAC	CTTCACAGAGCAATGACT CCAAAG
NOS2	GGCAGCCTGTGAGACCTTTG	GCATTGGAAGTGAAGCGTTTC
COX2	GGGTTAAACTTCCAAAGGAGACATC	CAGCCTGGCAAGTCTTTAACCT

## Additional Points

The English in this document has been checked by at least two professional editors, both native speakers of English. For a certificate, please see the following: http://www.textcheck.com/certificate/gLdxYL.
